# Hardox 450 Weld in Microstructural and Mechanical Approaches after Welding at Micro-Jet Cooling

**DOI:** 10.3390/ma15207118

**Published:** 2022-10-13

**Authors:** Abílio P. Silva, Tomasz Węgrzyn, Tadeusz Szymczak, Bożena Szczucka-Lasota, Bogusław Łazarz

**Affiliations:** 1 Department Electromecanica, Universidade da Beira Interior, Rua Marquês d’Ávila e Bolama, 6201-001 Covilhã, Portugal; 2 Faculty of Transport and Aviation Engineering, Silesian University of Technology, Krasińskiego 8, 40-019 Katowice, Poland; 3 Motor Transport Institute, Jagiellońska 80, 03-301 Warsaw, Poland

**Keywords:** welding, micro-jet, Hardox, high-strength steel, joint, characterisation, parameters, microstructure, fracturing, quality

## Abstract

The demand for high-strength steel welds, as observed in civil and transport engineering, is related to a mass reduction in vehicles. Container-type trucks are examples of this kind of transport means because their boxes are able to be produced using Hardox grade steels. Therefore, this study reflects on the properties of welds in the MAG welding of Hardox 450, obtained through an innovative micro-jet cooling process with helium. This joining technology aims to reduce the formation of defects and to obtain a joint with very good assumed mechanical properties. Structural components of grade steel require welds with acceptable mechanical parameters with respect to operational loading conditions. That is, this study focuses on selecting welding parameters for the Hardox 450 steel and determining the weld quality with respect to microstructural observations and mechanical tests, such as the Charpy, tensile and fatigue tests. Weld fracturing under increasing monotonic force was examined and was strongly related to both stress components, i.e., axial and shear. The joint response under fatigue was expressed through differences in the fracture zones, i.e., at a stress value lower than the proportional limit, and weld degradation occurred in the shear and axial stress components. The data indicate that the hourglass specimen, with the weld in the centre zone of the measurement section, can be directly used to determine a weld response under cyclic loading. The impact test results showed attractive behaviour in the tested joint, as represented by 47 J at −20 °C. The recommended MAG welding parameters for Hardox 450 steel are low-oxygen when using an Ar + 18% CO_2_ shielding mixture. The collected results can be directly used as a guide to weld thin-walled structures (6 mm) made of Hardox grade steel, while the data from mechanical tests can support the modelling, designing and manufacturing of components made from this kind of steel grade.

## 1. Introduction

Modern structures and vehicles are used in a variety of areas of industry, e.g., automotive, civil and transport, to improve standards related to the daily functioning of many communities [1,2,3]. This can be conducted through the application of new materials, which offer attractive resistance to mechanical loading in response to operational conditions, as compared to typical ones. In this case, a final product with different types of bodies and special vehicle bodyworks were manufactured to cover container-type truck boxes [4,5,6,7,8] or dumper truck boxes, as well as a dustcart. This was possible by reducing the number of supports/components of the vehicle’s body [9,10] and by applying high-strength steel, such as Hardox grade steel. This kind of material was also applied to the construction of concrete mixers. In this case, the application could be conducted using Hardox 450 high-strength steel [11,12,13,14].

This material has also been classified as low-alloy wear resistant cast steel and is therefore recommended for the following applications: Screen plates, lining, buckets, cutting edges and hoppers [15]. This steel grade has also been used for the wear plate, which ensures the maximum payload and a longer service life [16]. 

In comparison to typical structural materials, the application of Hardox steel grade enables a reduction in wall thickness of 35%. Using this approach, a mass reduction can also be reached. Nevertheless, it is worth emphasising that a Hardox grade higher than 450 should not be applied in salt water due to the increased risk of hydrogen cracking [17]. Generally, Hardox steel has the following advantages [18]:

- longer service life,
- made for the most challenging abrasive environments,
- high values of yield stress and ultimate tensile strength,
- reduced cost of production, maintenance and repairing,
- high resistance to impact, and
- uncompromising through–thickness hardness.

These steels are difficult to weld [19,20] because cracks can occur in joints and in certain other regions, leading to local fracturing and decohesion. The weldability tests (Węgrzyn since 1999) for Hardox 450 steel enabled the classification of welding processes regarding various oxygen levels and the collection of low-alloy steels. The most important criterion was the joint-absorbing energy measured in the Charpy’ impact test. In the case of Hardox 450 high-strength steel (from a different material group), it was decided to check the behaviour of the joint made with various oxygen processes in impact and fatigue tests. This approach enabled us to extend the knowledge concerning differences in energy at fracturing, which was necessary to estimate the occurrence of cracks. Due to there being frequent defects and irregularities after welding Hardox steels, a micro-jet helium cooling process during the MAG welding process was used. The micro-jet technology has been successfully proven in the welding of low-alloy steels and AHSS steels due to the formation of a fine structure in the weld. 

Concerning the wide application of Hardox steel, this grade of material and its welded joints were examined to determine mechanical resistance under various types of loadings. Other than typical mechanical experiments, a tensile test is a fundamental tool [8,21,22], in addition to others connected with cyclic loading because it enables us to follow material behaviour at various loading parameters up to fracture [23,24]. Fatigue data can be represented by the Wöhler curve at the cyclic rotational bending at a stress ratio equal to −1. It provided a fatigue limit value, represented by 450 MPa. For engineering applications and modelling approaches, this value can be compared to ultimate tensile strength, providing a value of 0.29. Another experimental procedure concerning high-strength steel grades, including the Hardox type, is tension–compression tests at R = −1 and at a frequency equal to 20 Hz. This test focuses on the number of cycles before fracture at cycle values ranging from 2 × 10^6^ to 2 × 10^9^. The proportion of stress amplitude and ultimate tensile strength was analysed and provided a ratio value between 0.2 and 0.9 at values of the mechanical parameter from 300 MPa to 2600 MPa [25]. 

### Details for Calibration of the Welding Technology

When welding the steel, a reduction in the mechanical parameter values in HAZ (Heat Affected Zone) can be Observed. During the Hardox steel welding process, it is recommended to limit the linear energy to the level of 5 kJ/cm [8]. Table 1 shows the mechanical properties of Hardox 450 steel used in the construction of shipping containers.

For this type of material, a yield stress value of 1200 MPa is obtained. This can be explained as a result of a higher carbon content and the presence of boron in the Hardox steel in relation to steels from the carbon–manganese group. In unalloyed B, it is rarely introduced and does not exceed 0.002% (Table 2).

The table data shows that the boron content is at a high level and significantly exceeds its presence in classic unalloyed structural steels. Boron is an effective deoxidizer and nitrogen-binding element. The boron content of the steel has a positive effect on the impact toughness of the joint. Boron also increases the strength of the joint. B is often introduced into the weld metal, together with Al and Ti. The aluminium content in the MAG steel weld metal should not exceed 0.03%. The Japanese patent owned by the Nippon Steel Corporation [27] contains information about the low impact toughness of the basic electrode weld metal containing 0.12% Al. 

However, the American patent [28] owned by the Incorporated Teledyne company shows that the content of Al in the weld metal of basic electrodes cannot exceed 0.03%. The research results obtained by G.M. Evans confirm this [29,30,31]. It is believed that aluminium binds oxygen more efficiently than titanium binds nitrogen [32,33]. 

Titanium and boron introduced simultaneously impact the effective binding of oxygen and nitrogen, which increases the impact toughness of joints made from various welding processes. Titanium and boron added to the weld metal in more significant amounts (over 0.02 of Ti and 0.002 of B) strengthen both the solution and precipitation of the weld metal deposit, which leads to an increase in the immediate, ultimate tensile strength. As a result, the hardness increases while the toughness of the weld metal decreases [34]. Masumoto [35] has confirmed that the optimal content of titanium and boron in the weld metal should be 0.01% to 0.04% Ti and 0.001 to 0.0025% B, respectively. Bonomo [36] has stated that, with higher amounts of Ti and B, the yield stress and ultimate tensile strength of the weld metal can theoretically be as high as 2500 to 4000 MPa/1% Ti. Widgery [37,38] confrimed that the self-reinforcement of titanium of an unalloyed joint containing 0.002% of B amounts to 60 to 70 MPa/1% Ti. Titanium and boron might enter the weld from the parent material and the electrode’s wires. The optimal boron content in the coated electrodes weld metal depends on not only the oxygen content, but also on the range of other elements, especially C, Al, Ti and N [39]. Reducing the content of C, Al, Ti and N in the weld metal allows for an increase in the content of B in the weld metal, because the total hardening and strengthening effect of the weld metal is lower. In the AWSE7018 electrode weld metal, the optimal content of titanium is from 0.002% to 0.07%. With the increase in titanium, the strength of the weld metal increases. The most favourable toughness is achieved with a Ti content level of 0.03%. At that time, the grain becomes refined and the oxygen content is reduced to the level of 400 ppm, which produces improved impact toughness. When manganese and silicon are decreased too much, and the appearance of titanium carbides and carbonitrides develops, the hardening of the weld metal and transformation of austenite into a bainite region can be observed.

Boron might also be added to coatings and fluxes in the form of B_2_O_3_ oxide. Boron dissolves in the weld metal as a result of this reduction. It can also be added in the form of ferro boron, or less frequently, in the form of B_4_C boron carbide [24,25,40]. According to the Nippon Steel Corporation’s patent GB20150337B, the basic electrode weld metal with 1.45% Mn is the most resistant to cracking in the CTOD (Crack Tip Opening Displacement) test, with a content of 0.03% to 0.05% of Ti and 0.005% to 0.007% of B simultaneously [41]. It is believed that a low concentration of boron promotes segregation of the boundaries of austenite grains and hinders ferrite nucleation. However, with an amount of boron greater than 0.005%, the impact toughness of the weld metal begins to decrease because Fe_23_(BC)_6_ carbide is precipitated at the austenite grain boundaries [42]. There is also the argument that it is possible to obtain a weld metal with high impact toughness at negative temperatures when strictly controlling the content of titanium, boron, nitrogen and oxygen in the weld metal [37]. It is, however, very difficult to implement this under average production welding conditions. G. M. Evans investigated the influence of boron on the properties of the unalloyed weld metal at different nominal Ti contents: 5, 35, 120, 260, 400 and 600 ppm [43]. He found that boron in the tested quantities, 1 to 195 ppm, did not reduce the oxygen concentration in the weld metal—unlike Ti—but changed the microstructure of the weld, thereby increasing the amount of acicular ferrite in it. At the same time, the amount of grain boundary ferrite with low impact toughness is reduced. In general, the toughness of the weld metal is the highest at the ratio Ti/B equal to 10 [44]. However, in the Hardox 450 high-strength steel, the boron content is twice as high as in unalloyed steels, and the Ti content is very low (Table 2). 

The formation of hard, non-metallic inclusions, especially BN and M_23_(C, B)_6_, which strengthen the weld metal and increase tribological properties, is much more advantageous. Until now, Hardox steels in a CO_2_ shield have very often been welded. This is associated with the possibility of crack formation in both the heat affected zone and in the weld itself. The authors of this publication believe that choosing this gas for welding Hardox 450 steel is inappropriate, and they decided to prove it. The oxygen content in the steel weld metal deposit made in the CO_2_ shield is at a high level (550 ppm), while the oxygen content in the weld metal deposit made in the pure argon shield is too low (approximately 280 ppm). The oxygen content in the alloy made in the Ar + 18% CO_2_ shield is at the most appropriate level (approximately 350 ppm). The classification of welding methods into various oxygen processes and their justification was carried out and published by one of the authors of this publication at the ISOPE-1999 Conference in France [45].

Hardox steel is a very modern structural material and is restricted concerning its welding process. Therefore, this article focuses on selecting the welding parameters for the Hardox 450 joint. The approach to the problem considered is represented by microstructural observations and mechanical tests, including monotonic tension, Charpy impact, bending and fatigue probes. These experiments were selected to collect the mechanical properties of the weld, which are important to model and qualify the joint to operation, i.e., proportional limit, yield stress, ultimate tensile strength, fatigue limit and impact absorbed energy. Compared to the MAG approaches, the biggest advantage of this paper is related to the welding parameter proposal, which provides desirable mechanical properties to the joint.

## 2. Details of Weld Manufacturing, Inspection and Testing

In the welding process of Hardox 450, 6 mm thick sheets were used. It was decided to prepare MAG (Metal Active Gas) joints by applying two different shielding gases in the tests: CO_2_ and the mixture of Ar + 18% CO_2_. Since both gases have different degrees of oxidation, it was decided to verify their influence on the weldability of the steel (gas mixtures according to the PN-EN 14175 standard). The shielding gas intensity was at the level of 15 L/min.

Two electrode wires were selected (see Table 3). The wire with a lower content of titanium was marked as T2 in the tests (EN ISO 14341-A: G42 3 C1 3Si1), and the wire with a higher content of titanium was marked as T4 (EN ISO 14341-A: G4S1). Both wires had a very similar chemical composition, except for their titanium content. The diameter of the electrode wire was 1 mm, the amperage was equal to 120 A, and the arc voltage reached 20 V. The parameters of helium micro-jet cooling were typical [8]. The diameter of the stream was equal to 60 µm and the gas pressure reached 0.6 MPa.

The chemical composition of the wires (Table 3) differed from that of the welded steel. It is worth noting that there was a lower carbon content in the wires than in the steel. Both electrode wires were selected in a way that ensured a difference in the titanium content, which, together with boron in the weld metal, affected the properties of the weld.

**Figure 1:**
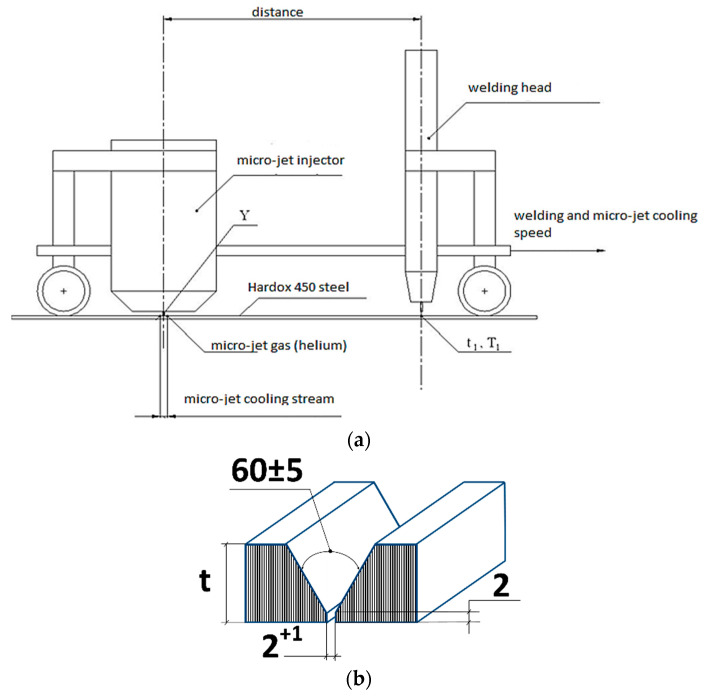
Scheme of the welding process with micro-jet cooling and (**a**) the groove shape and beveling method from the Hardox 450 steel with a thickness t = 6 mm (**b**).

The DC source was connected to (+) on the electrode and the thin-walled weld (6 mm) had a three-pass stitch. The welded joints were made from Hardox 450 steel, with a thickness of 6 mm in a flat position with V bevelling. The groove shape and the method of arranging subsequent layers are presented in Figure 1. The requirements of the EN 15614-1 standard were used for the MAG welding method at the low position (PA). The s total dimensions were 6 mm × 250 mm × 350 mm.

Non-destructive testing (NDT) was carried out to evaluate the quality of the obtained joints. This was checked as to whether the welded joint had welding defects, such as cracks and bubbles. Visual tests (VT) of the welded joints obtained by welding with micro-jet cooling were made with the eye, fitted with a magnifying glass at 3× magnification. This test was performed according to the PN-EN ISO 17638 standard and the assessment criteria according to the EN ISO 5817 standard. Moreover, the magnetic-powder tests (MT) were carried out by the PN-EN ISO 17638 standard. The assessment criterion was according to the EN ISO 5817 standard, using a magnetic flaw detector device type REM–230.

The test results are listed in the tables. Specimens that passed the test were subjected to tensile and bending tests.

A bending experiment was performed for all the joints in which no welding defects were detected. The test specimens had a cross-section of 6 mm × 30 mm. A mandrel with a diameter of 100 mm, at a bending angle of 180°, was used in the experiment.

A tensile test was carried out based on the PN-EN ISO 6892-1: 2020 standard. The experimental approach was designed to follow the Hardox 450 weld behaviour under static and fatigue loading. A tensile test was carried out based on the regimes of the PN-EN ISO 6892-1: 2020 standard.

The specimens that had the best results in the tests were taken for microstructural tests.

The next stage of the research included examinations of the microstructure of the specimens digested with the Adler reagent using a light microscope (LM). The study aimed to assess the microstructure and to compare the microstructures of specimens obtained in the welding process with micro-jet cooling and made at different process speeds. For specimens with the most favourable microstructures, the hardness of the welded joint was measured. Hardness measurements were completed in accordance with the guidelines of the PN-EN ISO 9015-1: 2011 and PN-EN ISO 6507-1: 2018-05 standards. A hydrogen test was performed for the same specimens. Tests on the content of H in the weld were carried out according to the glycerin method described in the standard “Determination of the total amount of hydrogen in the weld metal of steel electrodes with acid, rutile or alkaline coating. BN-64/4130 (BN-64/4130, 2013)”. Based on the above tests, the best connector was selected in respect of the quality of the structure and its properties. A fatigue test was also performed for such a selected connector. The probe collecting specimen design was performed in accordance with the ASTM E468-18 standard. This kind of experiment was chosen because of its wide ranging assessment of the joints under various levels of stress, i.e., high and low values, and because it collects the fatigue limit values. In this way, the joint behaviour can be captured, and many details on fracturing can be noticed. This means that the results can not only be used in material sciences, but can also be used in stages of the designing and inspection process and for modelling and durability prediction. It is worth noting that, from an experimental point of view, in the case of sheets, the preparation of specimens for other kinds of tests, such as impact and bending tests in compliance with their requirements, is not possible. Therefore, this kind of experiment has a significant role in the assessment of joint quality.

A bending test was performed for all the joints without welding defects. The cross-section of the specimen was 6 mm × 30 mm. A mandrel with a diameter of 100 mm, at a bending angle of 180°, was used in the experiment.

A tensile test was carried out in accordance with the PN-EN ISO 6892-1: 2020 standard. The experimental approach was designed to follow the Hardox 450 weld behaviour under static and fatigue loadings. Therefore, the U-notched specimen was designed. This was completed based on the requirements of the E 468–90 ASTM standard for fatigue tests. This type of specimen, with respect to the U-shape in the middle of the measuring zone (as the place of the weld), only enabled this area to be tested because it had the highest stress values. This means that the development of damage, regardless of the type of loading (static and fatigue), will occur in the weld, excluding areas such as HAZ and the fusion line. The shape of the specimen is especially recommended for testing narrow-width joints. Advantages of this specimen kind can be easily noticed, as compared to flat specimens, which do not offer a localisation of a maximum value of stress in a weld, but rather in a random region of a measuring section and even omits the joint. However, in the case of the U-notched specimen, the weld is directly located in the thinning section, thus causing the occurrence of the maximum stress value. In this way, the weld material, HAZ, and fusion line are directly examined in terms of the quality of the joints as a whole. This means that it is not necessary to reveal the position of the weld. The only condition required to manufacture the U specimen is to use an appropriate technological process in which part of the main axis of the weld and the horizontal axis of the measurement zone of the specimen are in line.

All the specimens were manufactured using the production technology applied in a specific steel structure production plant. These included both cutting and machining. Therefore, the tested specimens have scratches on their measuring surfaces.

**Figure 2:**
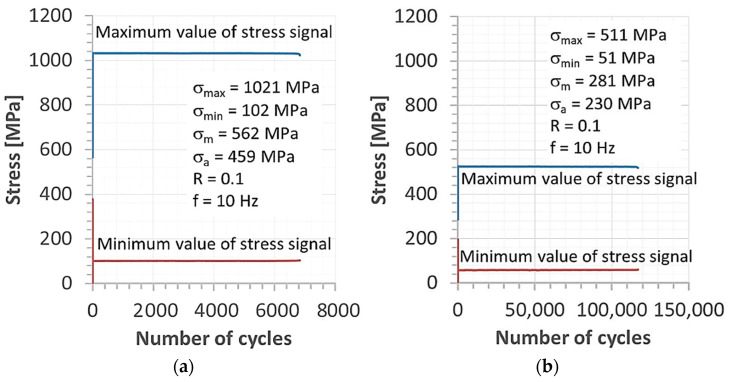
Levels of maximum and minimum values of stress signal versus the number of cycles used to control the testing machine for the fatigue process: (**a**) at high and (**b**) low-stress levels.

A fatigue test design to collect the specimen was performed in accordance with the the ASTM E468-18 standard. This kind of experiment was chosen because of its wide range of assessments of the joints under various levels of stress, i.e., high and low values (Figure 2), and because it collects the fatigue limit value. In this way, the joint behaviour can be captured, and many details of the fracturing can be noticed. 

This means that the results can not only be used in material science, but also in stages of the design and inspection, as well as in the modelling and durability prediction. It is worth noting that, from an experimental point of view, in the case of sheets, preparation of the specimens for other kinds of tests, such as impact and bending tests in accordance with the requirements, is not possible. Therefore, this kind of experiment has a significant role in assessing joint quality. 

**Figure 3:**
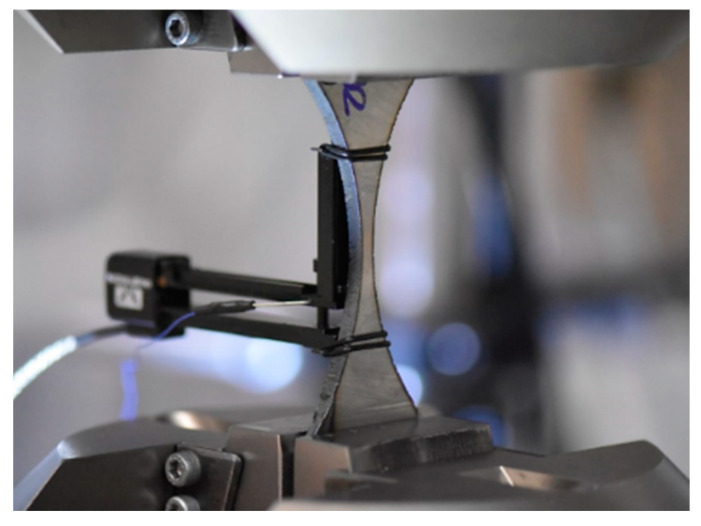
U-notched specimen and the 2620-601 extensometer in the 8802 Instron testing machine before the tensile test.

An additional tensile test (Figure 3) was selected to determine the material behaviour by employing the hourglass specimen because of the fatigue experiment. The test was carried out up to the material fracture and captured changes in axial strain values. The final stage of the test was represented by photos that reflect the measurement section and fracture zones in both perpendicular views.

The fatigue test was supported by the requirements of the ASTM E468-18 standard [46] and employed the shape of the specimens and a value of the radius for the necking. This kind of hourglass specimen was elaborated on by one author of the article [47,48] in regards to application to the joints. The characteristic features of the specimen’s geometry are connected to a weld region, which directly occurs in the middle sections of the measuring zone. In this respect, the joint is subjected to loading without any disturbance of other areas. Moreover, observations of the welding behaviour under fatigue cycles were directly conducted, focusing on fatigue damage occurrence and how it increases up to the cracks and material separation. Therefore, this kind of specimen was used. The fatigue signal was represented by a sinusoidal function at a stress ratio equal to 0.1 and a frequency of 10 Hz (Figure 3). The stress levels were selected to analyse the tensile curve and mechanical parameters. They were used to indicate the maximum stress values used, which were chosen following the tensile curve and values of mechanical parameters. 

## 3. Results and Discussion

### 3.1. The Weld in Non-Destructive, Bending and Impact Tests

The best results of the NDT (Table 4 (a)) can be achieved when the shielding gas mixture of Ar + 18% CO_2_ is applied. The combined effect of the boron appearing in the steel with a too high concentration of titanium from the wire is disadvantageous. Welding at a lower speed provides better results. No welding defects and non-conformities were found in the specimens: Sa1, Sa2, Sa3, Sa5 and Sa6 (Table 4 (b)).

The bending test only had positive results in cases where micro-jet cooling was introduced into the MAG welding process (Table 4 (b)). Therefore, it was decided to only continue testing connectors made using the micro-jet process.

The joints showed no defects after an attempt to bend them through at an angle of 180°. For further impact tests, it was decided to also consider those specimens in which no defects and non-conformities were found (Sa1, Sa2, Sa3, Sa5 and Sa6), as in the bending tests. 

The joint under the Charpy impact test was assumed to have acceptable plastic properties when the impact strength had a contractual value of 47 J at the lowest possible temperature. Impact accumulated energy at a minimum level of 47 J at a temperature of −20 °C—it is the so-called second class of toughness. This means that at any temperature higher than −20 °C, the accumulated energy is above 47 J, the joint is properly made and it is in the second quality class, which is required for joints made of high-strength steel. The results from the impact probe at a temperature of −20 °C are presented in Table 5 (a). They are in the form of average values at three measurements.

The results indicate that the second class of impact toughness minimum 47 J at −20 °C achieved only two welds and were made in the shielding mixture of argon and carbon dioxide (less oxidizing shielding gas). The impact resistance of the joint manufactured at a lower welding speed is higher than the others. Therefore, for further research, the joint marked as Sa1, made in the shielding mixture of Ar + 18% CO_2_, with the wire with a lower titanium content (0.002%) and welded at a speed of 300 mm/min, should be selected. For a complete understanding of the fracture energy of the specimens, additional impact toughness tests were carried out at temperatures below and above −20 °C, i.e., at −10 °C and at −30 °C. The results are presented in Table 5 (b).

At the temperature of −10 °C, all the joints had good plastic properties and fulfilled the first class of impact toughness, which proves that they had well-chosen welding parameters. It is worth noting that the most critical information for acceptance of the welding technology is the result of the Charpy test at the lowest possible temperature. Therefore, it was decided to analyse the fracture energy of the specimens at −30 °C. In this case, it was confirmed that the Sa3 and Sa5 welds had the best plastic properties. The third impact strength class (impact strength over 47 J at −30 °C) will only be met by one Sa1 weld (Table 5 (b)).

### 3.2. Microstructure and Hardness of the Weld

**Figure 4:**
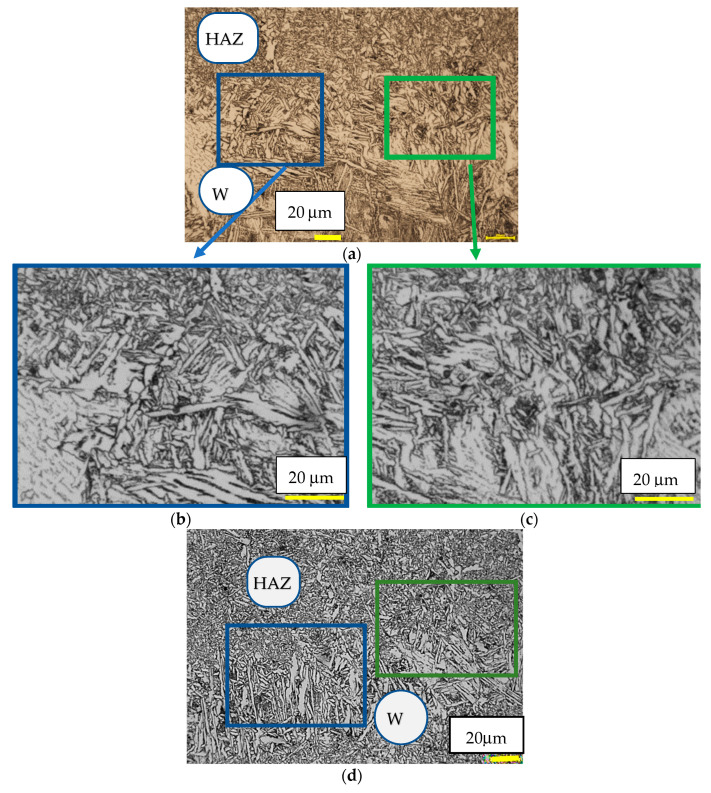

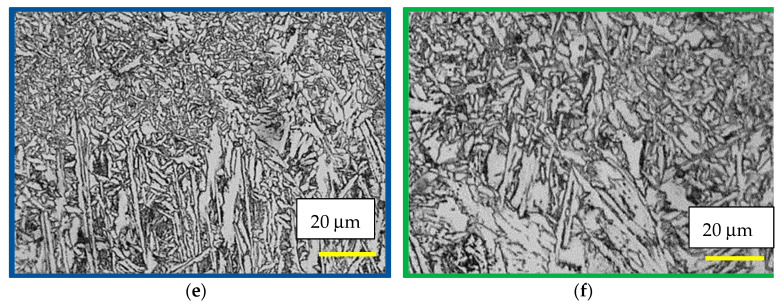
Microstructure of welds made with various process speeds (welding with micro-jet cooling), observation at different magnifications, (**a**–**c**) specimen Sa1 (300 mm/min), LM (**d**–**f**) specimen Sa5 (450 mm/min), and LM (W—weld material, HAZ).

The metallographic joint microstructure was also observed for the fractured regions due to the impact test. A specimen was etched with Adler’s reagent. Figure 4 presents the microstructure of the Sa1 weld (made in a shielding gas mixture of Ar + 18% CO_2_) and Sa4 weld (made in a shielding gas, CO_2_).

**Figure 5:**
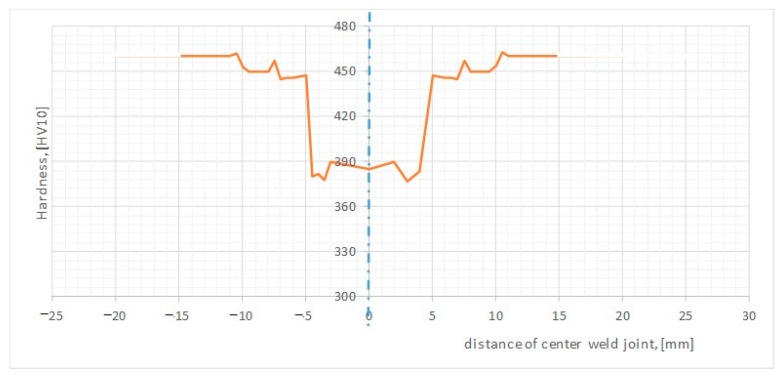
Hardness changes in the welded joint of Hardox 450 steel for specimen Sa1.

The performed qualitative evaluation of the examined joints showed that welding with micro-jet cooling led to favourable microstructural changes within the entire joint (Figure 4a–f). The microstructure of the welded joint of Hardox 450 steel, in the fusion zone, indicates a weak outline of the fusion area. In the heat affected zone, a structure of post-martensitic orientation with areas of bainite and troostite is visible. The observed microstructure is comparable to the structure presented in the literature [49,50]. The observation of the base material zone confirms that, after welding with micro-jet cooling, the material microstructure of the base material (BM) showed features characteristic of martensite and was not the tempered sorbite. Sorbit is obtained for Hardox joints after welding without micro-jet cooling, which is confirmed in Reference [8]. Martensitic structures for the joint of the Hardox steel are mainly obtained after welding and subsequent heat treatment. The micro-jet cooling process affects the structure of the welded material, thus preventing loss of its high mechanical properties (Figure 5). In the welded metal zone, directly after welding, microstructures typical for variable temperatures and cooling rates are observed. The microstructure of the joint is composed of:

- martensite and tempered martensite with areas of upper bainite (Figure 4b,c); and
- martensite and tempered martensite with areas of upper bainite with course ferrite (Figure 4f).

Therefore, in the author’s opinion, to obtain a high-quality welded joint with mechanical properties correlated with parameters of the base material, the newly developed welding with micro-jet cooling should be used. The results show that the newly developed welding with micro-jet cooling operation led to favourable structural changes within the entire joint.

It is visible that, in the Sa1 specimen, made in a shielding gas mixture of Ar + 18% CO_2_ with helium micro-jet cooling and lower process speed, tempered martensite and bainite are treated as the dominate structures. In the Sa5, made with helium microjet cooling and a higher process speed, apart from martensite and bainite, a small amount of course ferrite was observed. Both structures (Sa1 and Sa5) were rather similar to that of the base material, where martensite was a dominant structure. It was proven that micro-jet helium cooling is recommended to obtain welding joints of good quality. It was shown that using a gas mixture containing argon instead of pure carbon dioxide as a shielding gas prevents the formation of ferrite in high contents. Ferrite, especially in its overgrown form, could seriously reduce the mechanical properties of the welded material. It can be noticed (Figure 4) that an important welding parameter for Hardox 450 steel is the process (welding with micro-jet cooling) speed, which allows slightly different structures that are not dominant to be obtained. 

It was noticed that the microstructure of the Sa1 and the Sa5 welds were very similar (Figure 4) and this indicated that the speed value of the welding process, i.e., 300 mm/min and 450 mm/min, created the same (tempered martensite microstructure with areas of upper bainite). The minimal amount of course ferrite (in W) and troostite (in HAZ) slightly changed the joint structure of the SA5 specimen, so slightly lower hardness values were recorded in the heat affected zone in the specimen SA5 and were comparable in the weld (W). Overall, the hardness distribution was similar in both joints. For this reason, only one type of tested weld (Sa1) was qualified for further testing. The selected joint showed slightly better properties in the bending and impact tests and it was included in Section 3.1 of the article presented. The hardness results of the tested specimens SA1 are additionally shown as a graph (Figure 5) to approach the mechanical resistance of the joint zone on loading.

Microscopic studies did not show any joint failures in the form of cracks. An overview of hardness changes in the welded joint of Hardox 450 steel is presented in Figure 5. The material Vickers hardness in the BM zone was about 450 MPa after welding with micro-jet cooling. The strength properties of the base material were not lowered due to the welding process used. In the central part of the weld metal, a slightly reduced hardness of 390 MPa was noticed (Figure 5). The hardness drop can be explained by the chemical composition of the weld metal. The composition in the weld is significantly different from the composition of the base material, and it affected the properties after welding. The maximum hardness in the heat affected zone was 451 MPa. The minimum hardness of the weld material was 375 MPa.

### 3.3. Hydrogen Diffusion

The next stage of the research was to estimate the diffusible hydrogen content for the two tested joints, which in the previous study were characterized as having the best properties (S1 and S5). Immediately after welding, the diffusible hydrogen content in the weld was checked. The test results are presented in Table 6.

Tests of the content of H in the weld were carried out according to the glycerin method described in the standard “Determination of the total amount of hydrogen in the weld metal of steel electrodes with acid, rutile or alkaline coating. BN-64/4130 (BN-64/4130, 2013)”. Based on the measurements obtained through the glycerin test, the value of hydrogen was estimated, which could be obtained in the mercury test, using the well-known formula presented in Reference [45].

Equation (1) allows for the determination of the relationship in the following forms: Diffusible hydrogen amount in the deposited metal from the glycerin method and diffusible hydrogen amount in the deposited metal from the mercury method [51]:

HD_glyc_ = 0.789 × HD_merc_ − 2.13 [mL/100 g] (1)

where:

HD_glyc—_diffusible hydrogen amount in the deposited metal from the glycerin method (mL/100 g Fe),
HD_merc_—diffusible hydrogen amount in the deposited metal from the mercury method (mL/100 g Fe).

Based on the results of the tests presented in Table 5, it was found that the diffusing hydrogen content in both welds was at the recommended level of 3–7 mL/100 g of the weld metal. These low diffusible hydrogen levels in the weld show that the selected process was correct.

### 3.4. The Weld Behaviour in Static and Fatigue Tests

**Figure 6:**
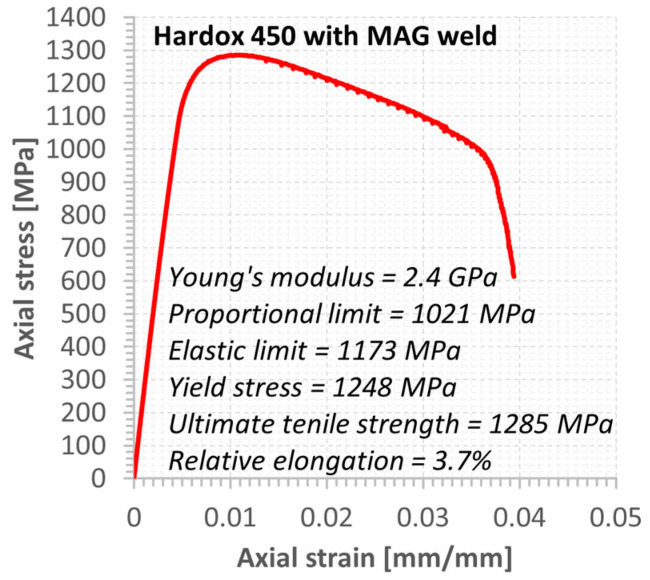
Tensile characteristics and mechanical parameters of the Hardox 450 MAG weld.

**Figure 7:**
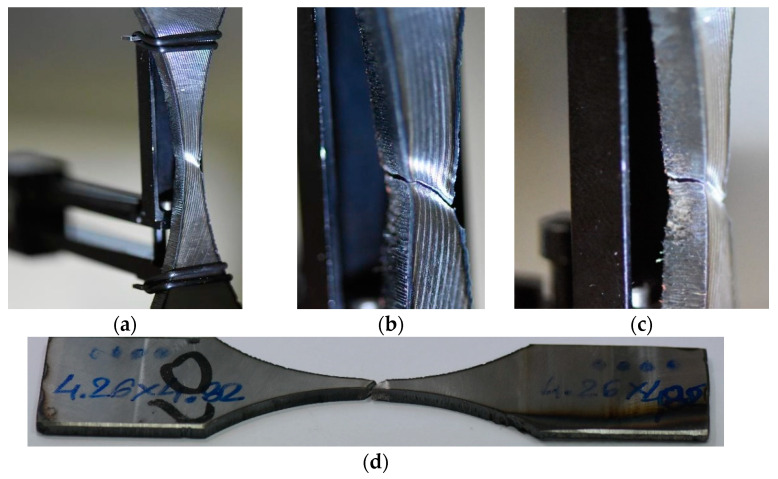
Hardox 450 MAG weld at the final stage of the tensile test: (**a**) General view, (**b**,**c**) fracture region, and (**d**) the specimen out of the testing machine.

**Figure 8:**
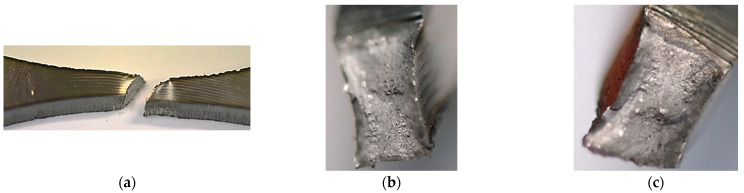
Fracture zone of the Hardox 450 welded in MAG process after the tensile test: (**a**) general view, and (**b**,**c**) both parts of the region.

The behaviour of the Hardox 450 with the MAG weld reflected elastic and elastic-plastic responses with a prolonged instability region (Figure 6). This expresses that weld fracturing occurs at a tri-axial stress state. Moreover, more details on the last stage of the specimen under tensile force can be noticed based on photos (Figure 7 and Figure 8). They can be selected based on the orientation of the fracture region, in which edges are sloped to the main axis of the specimen. This feature indicates that weld degradation appeared under axial and shear stresses to the fracture plane. It should be noted that the obtained results are global data that lead towards the welded joint without considering the HAZ and fusion line. Therefore, despite the use of the U-specimen, which concerned the position of the fracture plane at an angle, the obtained results indicate the significance of the -zones mentioned above in the description of the weld fractography. 

The fatigue process of the weld was observed at a stress range between 255 MPa and 1180 MPa (Table 7). This shows the significant differences between the maximum and minimum values of the stress used as an effect of the MAG weld occurrence. In this case, the ratio was represented by 4.7, which reflects the sensitivity of the Hardox 450 to the welding process applied. These data have also enabled us to approach the fatigue limit using the Basquin’s equation (σ = 7792.25 n^−0.23^), with 298 MPa as the fatigue limit. This value was confirmed by the equation and by the fatigue data collected at 255 MPa and 2.79 × 10^6^ cycles without fracture. The fatigue limit value was used for the relationship between the mechanical parameter of the weld and a proportion value was calculated (Table 8). This kind of data follows engineering efforts in regards to practical aspects of designing and diagnostics because it reflects specific values (Table 7). 

**Figure 9:**
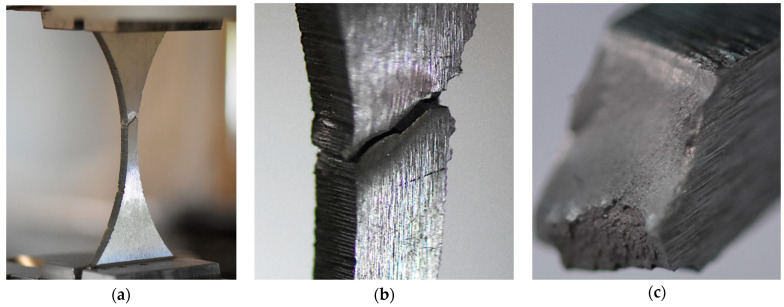
Hardox 450 with the MAG weld from the fatigue test under the maximum value of stress equal to 1021 MPa, (**a**,**b**) directly after separation, and (**c**) fracture region.

In the case of a stress value equal to the proportional limit, the weld degradation was represented by the fracture plane located at an angle; providing the axial and shear stress are essential for joint degradation. For this, it can be concluded that the tested region separation occurred in the biaxial stress state as a result of the one-axial cyclic loading and its value (Figure 9).

**Figure 10:**
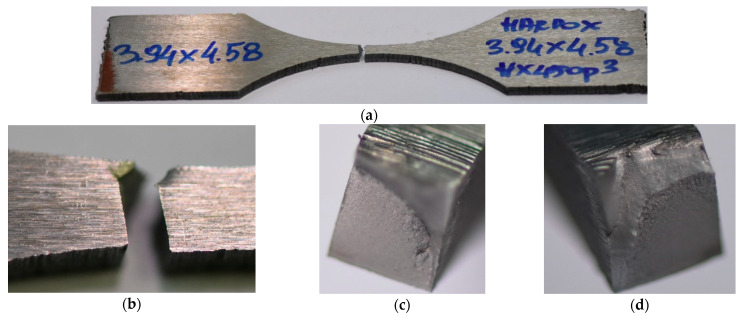
Hardox 450 with MAG weld from the fatigue test under the maximum value of stress equal to 511 MPa, (**a**,**b**) directly after separation, and (**c**,**d**) fracture region.

**Figure 11:**
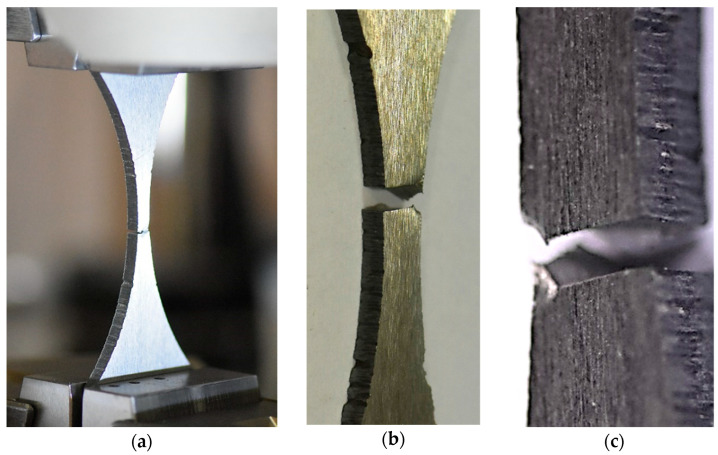
Hardox 450 with MAG weld from the fatigue test under the maximum value of stress equal to 340 MPa, (**a**,**b**) directly after separation, and (**c**) fracture region.

Different responses to the cyclic loading were noticed as the stress reached half the value of the proportional limit (Figure 10). This was expressed by the fracture zone features, such as the perpendicular plane to the main axis of the specimen and the more dominant fatigue region. This has shown that axial stress is a fundamental component of weld degradation under the stress considered. The same sentence can be formulated for data at the stress level of one-third of the proportional limit (Figure 11). The results can be summarized as the fracture of the Hardox 450 MAG weld the under cyclic stress value below the proportional limit being mainly caused by axial stress. 

**Figure 12:**
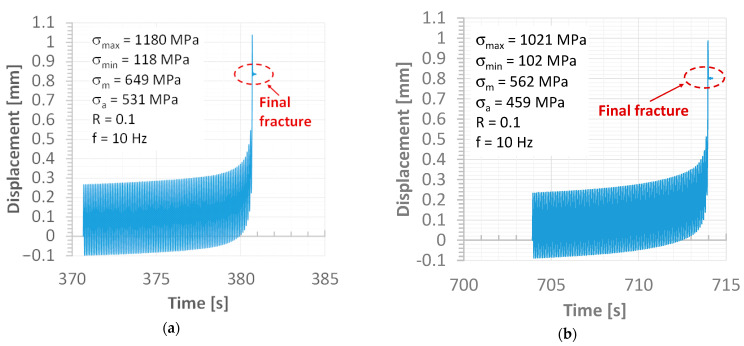

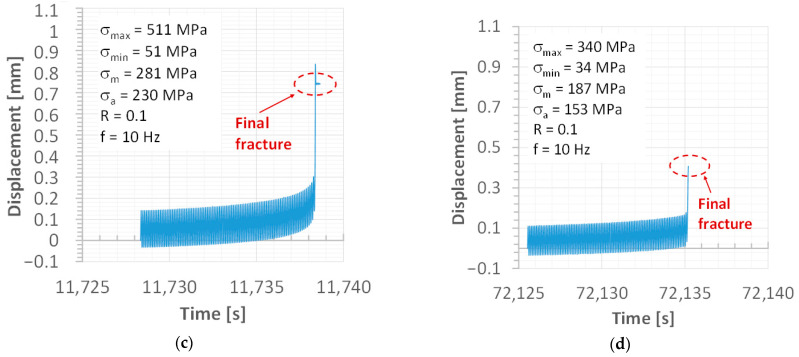
Displacement versus time at the finale stage of the fatigue test for the following values of maximum stress: (**a**) 1180 MPa, (**b**) 1021 MPa, (**c**) 511 MPa and (**d**) 340 MPa.

**Figure 13:**
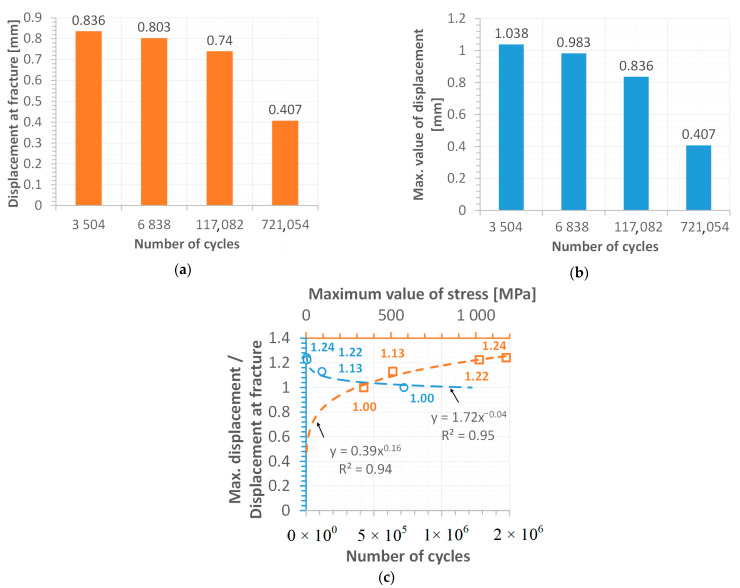
Variations of displacement (**a**), (**b**) its proportion versus the number of cycles and (**c**) maximum value of the stress.

Displacement values directly before and at fracture have reflected differences in the weld response. At the maximum value of stress following the elastic limit, the displacement value of 0.85 mm represented the specimen separation, however, (a) in contrast, for the stress at the proportional limit, the fracturing occurred at 6.3 % smaller values (b). The reduction in the displacement value was also observed at a stress level of half of the proportional limit (c) and its 33% value (Figure 12d and Figure 13a). It can be concluded that plastic features of the weld disappear when stress levels decrease. This sentence is also confirmed by details of the fracture regions, which expressed changes in the weld degradation from brittle–ductile (Figure 9 and Figure 10) to brittle (Figure 11) at the high and low values of stress used, respectively.

An analysis of the data relating to displacement has also indicated differences between the maximum value of the magnitude taken (Figure 12 and Figure 13b) and its value at the fracture evaluated, tending to a total reduction in its proportion at the stress level significantly below the proportional limit (Figure 12d and Figure 13c). As can be noticed, this proportion tends asymptotically to its limit level, which is expressed by a value of 1.0 with an increasing number of cycles. Concerning the stress levels used in the fatigue tests, this proportion follows a value close to 1.25 at higher ranges of the loading applied (Figure 13c).

## 4. Summary

Concerning the Hardox 450 weld quality, it was decided to check what is the most appropriate electrode wires and gas mixtures based on their chemical composition, which affects the structure and properties of the welds. For the first time, innovative micro-jet cooling was used for the MAG welding of Hardox steel. Preliminary studies confirmed the validity of micro-jet cooling and much better results were obtained.

Electrode wires with a titanium content at the level of 0.002–0.004% were used for the welding process because titanium can enter the weld from the electrode wires, causing an increase in temporary strength. It also affects the microstructure, causing it to become refined. Through this, we can consider the value of the ultimate tensile strength of the weld material using the general formula for the structural steels as follows: Ultimate Tensile Strength = 3.5 Brinell Hardness. In this case, the joint’s UTS reached 1300 MPa and converted the average value of the Vickers (390 MPa) hardness to the Brinell (371 MPa) one. This shows that the value of the UTS of the joint was very similar to the UTS of the base metal, thus indicating a high quality of the manufactured weld [26]. Microscopic tests have confirmed changes in the microstructure in particular zones of the welded joints (base material, weld and heat affected zone). The light microscopy observation reflected that, after welding with micro-jet cooling, the material microstructure of the base material (BM) showed features characteristic of martensite. In the heat affected zone, the microstructure of post-martensitic orientation with areas of bainite and troostite was observed. With appropriately selected cooling parameters and linear energy of the welding process, joints were obtained without welding defects and incompatibilities. Thus, the accepted assumptions for the execution of welded joints of this steel are correct.

Moreover, the observed changes in the material’s structure between correctly made joints most likely resulted from the change in the linear energy of the welding process. In the joint weld structure of specimen SA5, we can observe areas of ferrite, which were not identified in the structure of specimen SA1. Registered changes indicate that there are better properties of specimen SA1.

Based on the research results (non-destructive and destructive tests), we can confirm, that the application of the newly developed welding process with micro-jet cooling is beneficial. In the correctly obtained joints, the Vickers hardness measurement was high (approximately 450 MPa in the BM zone and about 390 MPa in the joint).

To obtain a correct and reproducible joint, the use of micro-jet cooling alone is insufficient. At the same time, attention should be paid to the different oxygen contents of the welding process. To this end, it is important to select additional materials, including the shielding gas mixture and the corresponding electrode wire. Thermodynamic factors were considered. It was decided not to use preheating, but the influence of the welding speed on the properties of the welded joint was checked. 

The analysis of data taken from the MAG welding process with the micro-jet cooling, microstructural approach, and static and fatigue experiments have contributed to solving the problem of the quality of the joining type for the hard-rusting steel (Hardox 450). The conclusions are as follows:

1. The welded steel fracturing under static tensile force is strongly related to both stress components, i.e., axial and shear, which directly causes the region to appear at the angle, as compared to the main axis of the specimen;
2. Weld examination under cyclic loading can be conducted using an hourglass specimen with this kind of joint in the middle section of the measurement region;
3. Behaviour of the joint under the fatigue process was significantly related to a value of the stress level because different details of the fracture zones were observed. In the case of a stress value being equal to and exceeding the proportional limit of the weld, the joint degradation appeared as the shear and axial stress components, while at a more minor one, axial stress was a dominant reason. This response indicated the brittle features that become more significant in fracturing at a low value of stress;
4. Concerning the ultimate tensile strength (UTS), the fatigue limit of the tested weld was approximately 4.5 times lower, i.e., 298 MPa, indicating a relationship between the parameters mentioned in the following form: Fatigue Limit = 0.23 × UTS;
5. Application of the MAG welded steel in the engineering area with respect to technical safety should be planned and take the following values for the mechanical parameters: 1000 MPa (proportional limit), 1250 MPa (yield stress) and 298 (fatigue limit for the complete reversed cycles);
6. It is possible to obtain joints in the second impact class, which means that the specimen breaking energy in the impact test is above 47 J at −20 °C;
7. For the proper welding of Hardox 450 steel, a low-oxygen welding process should be used, which in the case of the MAG process, should consider the use of the Ar + 18% CO_2_ shielding mixture; and
8. A less oxidizing shielding mixture in the MAG process will cause boron to bind with nitrogen to form BN nitride and titanium to bind with oxygen and to form TiO oxide. Thanks to this, the mechanical and tribological properties of the entire joint will have comparable properties.

## Figures and Tables

**Table 1:** Mechanical properties of the Hardox 450 steel [26].

Yield Point, YS [MPa]	Ultimate Tensile Strength UTS, [MPa]	Hardness, HBW [MPa]
1190	1355	450

**Table 2:** Chemical composition of Hardox 450 steel [26].

C, %	Si, %	Mn, %	P, %	S, %	Cr, %	Ni, %	Mo, %	B, %
0.26	0.7	1.6	0.025	0.011	1.4	1.5	0.6	0.005

**Table 3:** Chemical compositions of the electrode wires [6].

Wire	C%	Si%	Mn%	P + S%	Cr%	Ti%	Al%
T2	0.08	0.9	1.5	0.04	0.25	0.002	0.01
T4	0.09	1.0	1.7	0.04	0.15	0.004	0.01

**Table 4:** (**a**) The NDT test results of welded joints. (**b**) The results of the static bending test of joints with respect to cracks.

(**a**)					
**Mark**	**Shielding Gas Type**	**Electrode Wire Type**	**Welding Speed**	**Micro-Jet Cooling**	**Observations**
Sa1	Ar + 18% CO_2_	T2	300 mm/min	yes	no cracks
Sa2	Ar + 18% CO_2_	T4	300 mm/min	yes	no cracks
Sa3	CO_2_	T2	300 mm/min	yes	no cracks
Sa4	CO_2_	T4	300 mm/min	yes	cracks
Sa5	Ar + 18% CO_2_	T2	450 mm/min	yes	no cracks
Sa6	Ar + 18% CO_2_	T4	450 mm/min	yes	no cracks
Sa7	CO_2_	T2	450 mm/min	yes	cracks
Sa8	CO_2_	T4	450 mm/min	yes	cracks
Sa9	Ar + 18% CO_2_	T2	300 mm/min	no	no cracks
Sa10	Ar + 18% CO_2_	T4	300 mm/min	no	cracks
Sa11	CO_2_	T2	300 mm/min	no	cracks
Sa12	CO_2_	T4	300 mm/min	no	cracks
Sa13	Ar + 18% CO_2_	T2	450 mm/min	no	cracks
Sa14	Ar + 18% CO_2_	T4	450 mm/min	no	no cracks
Sa15	CO_2_	T2	450 mm/min	no	cracks
Sa16	CO_2_	T4	450 mm/min	no	cracks
(**b**)					
**Specimen Mark**			**Results**		
Sa1			no cracks		
Sa2			no cracks		
Sa3			no cracks		
Sa5			no cracks		
Sa6			no cracks		
Sa9			cracks		
Sa14			cracks		

**Table 5:** (**a**) The results of the Charpy-V impact test obtained at a temperature equal to −20 °C. (**b**) The results from the Charpy impact test, collected at temperatures between −10 °C and −30 °C.

(**a**)		
**Specimen mark**	**KV, J (−20 °C)**	
Sa1	54	
Sa2	42	
Sa3	45	
Sa5	50	
Sa6	44	
(**b**)		
**Specimen mark**	**KV, J (−30 °C)**	**KV, J (−10 °C)**
Sa1	48	67
Sa2	Below 30	53
Sa3	Below 30	57
Sa5	41	64
Sa6	Below 30	55

**Table 6:** Diffusing hydrogen content (mL/100 g of weld metal).

	Real H Content in Weld Obtained in Glycerin Method	Estimated Value of H in Weld that Could Be Obtained in the Mercury Method Using Fydrych Equation
S1	3.17	6.71
S5	3.29	6.86

**Table 7:** Proportion between the fatigue limit and the mechanical parameters of the Hardox 450 MAG weld.

Fatigue Limit/Proportional Limit	Fatigue Limit/Elastic Limit	Fatigue Limit/Yield Stress	Fatigue Limit/Ultimate Tensile Strength
0.29	0.25	0.24	0.23

**Table 8:** Stress and number of cycles from the fatigue test of the Hardox 450 welded in MAG.

**Maximum value of stress [MPa]**	1180	1021	511	340	225
**Number of cycles to fracture**	3504	6838	117,082	721,054	279,575

## Data Availability

Research conducted as part of the University’s BK-270-RT1-2022 projec.

## References

[1] Pawar N. Automotive Advanced High Strength Steel (AHSS) Market Professional Survey Report 2019. https://dataintelo.com/report/global-automotive-advanced-high-strength-steel-ahss-market/.

[2] Matlock D.K., Speer J.G., de Moor E. Recent AHSS developments for automotive applications: Processing, microstructures, and properties. Proceedings of the Addressing Key Technology Gaps in Implementing Advanced High-Strength Steels for Automotive Light Weighting.

[3] Varelis G.E., Papatheocharis T., Karamanos S.A., Perdikaris P.C. (2020). Structural behavior and design of high-strength steel welded tubular connections under extreme loading. Mar. Struct..

[4] Bleck W., Larour P., Baeumer A. (2005). High strain tensile testing of modern car body steels. Mater. Forum.

[5] Lahtinen T., Vilaça P., Peura P., Mehtonen S. (2019). MAG welding tests of modern high strength steels with minimum yield strength of 700 MPa. Appl. Sci..

[6] Sága M., Blatnická M., Blatnický M., Dižo M., Gerlici J. (2020). Research of the fatigue life of welded joints of high strength steel S960QL created using laser and electron beams. Materials.

[7] Jaewson L., Kamran A., Jwo P. (2011). Modelling of failure mode of laser welds in lap-shear specimens of HSLA steel sheets. Eng. Fract. Mech..

[8] Hadryś D. (2015). Impact load of welds after micro-jet cooling. Arch. Metall. Mater..

[9] Saravanan R. (2021). Design Using High Strength Steel, Knowledge and Support for Your Applications. Webinar.

[10] Szymczak T., Makowska K., Kowalewski Z.L. (2020). Influence of the welding process on the mechanical characteristics and fracture of the S700MC high strength steel under various types of loading. Materials.

[11] Porter D.A. Weldable high-strength steels: Challenges and engineering applications. Proceedings of the IIW International Conference High-Strength Materials-Challenges and Applications.

[12] Ma J.L., Chan T.M., Young B. (2017). Tests on high-strength steel hollow sections: A review. Proc. Inst. Civ. Eng. Struct. Build..

[13] Günther H.P., Hildebrand J., Rasche C., Versch C., Wudtke I., Kuhlmann U., Vormwald M., Werner F. (2014). Welded connections of high-strength steels for the building industry. Riv. Ital. Della Saldatura.

[14] Goritskii V.M., Shneiderov G.R., Guseva I.A. (2019). Effect of chemical composition and structure on mechanical properties of high-strength welding steels. Metallurgist.

[15] High Strength, Futeng Special Steel—Futeng Technology Company Ltd.. https://en.fimc.com.tw/high-strength.html.

[16] SSAB Product Program Sheet Metal, 813en-GB-SSAB Product program-V3-2012. Confetti. Spreadcard, Oxelösund Sweden. https://www.scribd.com/document/399639189/SSAB-Product-Program-Sheet-Metal.

[17] Reducing Environmental Impact with Hardox® Wear Plate—Now and in the Future, SSAB, Oxelösund Sweden. https://www.ssab.com/en/brands-and-products/hardox/sustainability.

[18] Piedra G. (2020). Advantages of Using Advanced High Strength and Wear Resistance Steels in Tippers and Trailers. https://www.motorindiaonline.in/wp-content/uploads/2020/05/SSAB-Presentation.pdf.

[19] Melloy G.F., Summon P.R., Podgursky P.P. (1973). Optimising the boron effect. Met. Trans..

[20] Chen H., Xue S.B. (1991). The transfer of small amounts of boron during SMA welding. Weld. J..

[21] Konat Ł., Białobrzeska B., Białek P. (2017). Effect of welding process on microstructural and mechanical characteristics of Hardox 600 steel. Metals.

[22] Mazur M., Ulewicz R. (2017). Analysis of strength and fatigue properties of constru0ction materials for manufacturing the parts of semi-trailers. Appl. Eng. Lett..

[23] Wang Z., Wu X., Liu D., Zuo X. (2021). Correlation between microstructure and fracture behavior in thick Hardox 450 wear-resistant steel with tin inclusions. Front. Mater..

[24] Ulewicz R., Szataniak P., Novy F. Fatigue properties of wear resistant martensitic steel. Proceedings of the 23rd International Conference on Metallurgy and Materials.

[25] Nový F., Bokůvka O., Trško L., Jambor M. (2019). Safe choice of structural steels in a region of ultra-high number of load cycles. Prod. Eng. Arch..

[26] Content Hardox® Guarantees, SSAB, Oxelösund Sweden. https://www.ssab.com/products/brands/hardox/products/hardox-450.

[27] (2009). Nippon Steel Corporation Steel Products for Welding. Patent.

[28] Teledyne Incorporated (1972). Coated Ferrous Low Hydrogen Arc Welding Electrode and Production of an Improved Nonaustenitic Steel Weld Deposit. Patent.

[29] Evans G.M. (1993). The effect of micro-alloying elements in C-Mn steel weld metals. Weld. World.

[30] Evans G.M. (1994). Microstructure and properties of ferritic steel welds containing Al and Ti. Oerlikon Schweissmitt.

[31] Evans G.M. (1982). Factors affecting the microstructure and properties of C-Mn all-weld metal deposits. Weld. Rev. Abroad WRC.

[32] Nakano S., Shiga A., Tsuboi J. (1975). Optimising the Titanium Effect on Weld Metal Toughness.

[33] Terashima H., Hart P.H. (1983). Effect of aluminium in C-Mn steels on microstructure and toughness of submerged arc weld metal, a progress report. Research Report 186/1982, 1982 and 65-th Annual AWS Convention.

[34] Beidokhti B., Koukabi A.H., Dolati A. (2009). Effect of titanium addition on the microstructure and inclusion formation in submerged arc welded HSLA pipeline steel. J. Mater. Process. Technol..

[35] Badri K., Narayanan L., Kovarik P.M., Sarosi M.A., Quintana M.J. (2010). Mills, Effect of microalloying on precipitate evolution in ferritic welds and implications for toughness. Acta Mater..

[36] El-Faramawy H.S., Ghali S.N., Eissa M.M. (2012). Effect of Titanium Addition on Behavior of Medium Carbon Steel. J. Miner. Mater. Charact. Eng..

[37] Widgery D.J. (1974). Deoxidation practice and toughness of mild steel weld metal. Rep. 2 Weld. Res. Int..

[38] Widgery D.J. (1976). Deoxidation practice for mild steel weld metal. Weld. Res..

[39] Zemlik M., Konat Ł., Napiórkowski J. (2022). Comparative Analysis of the Influence of Chemical Composition and Microstructure on the Abrasive Wear of High-Strength Steels. Materials.

[40] Simcoe C.R., Elsea A.R. (1955). Manning G.K. Study of the effect of boron in the decomposition of austenite. Trans. AIME.

[41] Nippon Steel Corporation (1981). Low-Hydrogen Coated Electrode. Patent.

[42] Masumoto H., Kobaayshi T., Watanabe K. (1965). Effect of Titanium Additions of Properties of the New Magnet Alloy “Malcolloy” in the. Co-Al System. Jpn. Inst. Met..

[43] Watanabe T., Nakamura H., Ei K. (1989). Grain Refinement by TIG Welding with Electromagnetic Stirring—A Study of Solidification Control of Austenitic Stainless Steel Weld Metal.

[44] Evans G.M. (1996). Microstructure and properties of ferritic steel welds containing Ti and B. Weld. J..

[45] Węgrzyn T. (1999). Classification of Metal Weld deposit in Terms of the Amount of Oxygen. Proceedings of the Conference of International Society of Offshore and Polar Engineers ISOPE´99.

[46] (2018). Standard Practice for Presentation of Constant Amplitude Fatigue Test Results for Metallic Materials.

[47] Szymczak T., Szczucka-Lasota B., Węgrzyn T., Łazarz B., Jurek A. (2021). Behavior of weld to S960MC high strength steel from joining process at micro-jet cooling with critical parameters under static and fatigue loading. Materials.

[48] Węgrzyn T., Szymczak T., Szczucka-Lasota B., Łazarz B. (2021). MAG welding process with micro-jet cooling as the effective method for manufacturing joints for S700MC steel. Metals.

[49] Frydman S., Konat Ł., Pękalski G. (2008). Structure and hardness changes in welded joints of Hardox steels. Arch. Civ. Mech. Eng..

[50] Teker T., Gencdogan D. (2021). Heat affected zone and weld metal analysis of HARDOX 450 and ferritic stainless steel double sided TIG-joints. Mater. Test..

[51] Fydrych D., Łabanowski J. (2015). An experimental study of high-hydrogen welding process. Rev. Metal..

